# Promoting Child Development During the COVID-19 Pandemic: Parental Perceptions of Tele-Home Visits in Early Head Start Programs

**DOI:** 10.1007/s10995-022-03520-4

**Published:** 2022-10-17

**Authors:** Delia Vicente, Melanie Venegas, Tumaini R. Coker, Alma D. Guerrero

**Affiliations:** 1 Development Behavioral Pediatrics Division, Department of Pediatrics, David Geffen School of Medicine, UCLA Early Head Start, University of California, Los Angeles, 14423 Van Nuys Blvd, 91331 Arleta, CA USA; 2 Department of Pediatrics, University of Washington School of Medicine, Seattle Children’s Research Institute, 1920 Terry Ave, 98108 Seattle, WA USA; 3 Department of Pediatrics, David Geffen School of Medicine, UCLA Mattel Children’s Hospital and Children’s Discovery & Innovation Institute, University of California, Los Angeles, 10833 Le Conte Avenue, 90095 Los Angeles, CA USA

**Keywords:** Early Head Start, Home visits, COVID-19, Telehealth

## Abstract

**Objective:**

The COVID-19 pandemic prompted families to receive Early Head Start (EHS) home-based services virtually. This qualitative study evaluated parental perceptions of EHS tele-home visits.

**Methods:**

EHS parents who had transitioned to tele-home visits using any video-chat platform were recruited to participate in a Spanish or English virtual focus group that assessed their perceptions of tele-home visits. Using an iterative, consensus-seeking inductive content analysis approach, themes and subthemes were identified.

**Results:**

Thirty-five mothers of children newborn to 3-years-old, where the majority were Latino and Spanish-speaking, participated in four focus groups. Several patterns pertaining to technology, child engagement, child learning and development, and parent-home visitor relationship emerged in the qualitative analysis. Mothers revealed varying degrees of digital proficiency, device preference, and technology challenges. Mothers reported variability in child engagement and concerns with missed socialization opportunities for children as a results of tele-home visits, but also reported increased self-efficacy in supporting child development, positive relationships with their home visitor, and overall satisfaction with services.

**Conclusion:**

Parents revealed tele-home visits have the potential to be a viable service delivery method for EHS home-based programs. While parents perceived increased engagement and an uncompromised parent-home visitor relationship, they revealed areas of needed support that would optimize the use of tele-home visits.

## Significance

*What is already known on this subject?* In-person Early Head Start home-based services have been associated with healthy child development and positive parenting behaviors. However, there are no known studies that have studied tele-home visits in Early Head Start. Research on the delivery of childhood intervention services via telehealth have reported both strengths and limitations.

*What this study adds?* This study adds to the literature valuable information on how tele-home visits are perceived by parents participating in Early Head Start home-based services. In this qualitative study on EHS tele-home visits, our findings suggest parents perceived an increased parental efficacy, engagement in monitoring their child’s development, planning with home visitors, and creating an engaging environment. Parents identified areas of needed support with child’s socialization needs, disengagement, and technological assistance. With support, tele-home visits have the potential to be a viable service delivery method for EHS home-based programs, particularly during the COVID-19 pandemic.

## Introduction

Early Head Start (EHS) programs are federally-funded child development programs that offer services to low-income families across communities in the United States (Fenichel & Mann, 2001). Since its inception in 1995, EHS programs have provided an array of services related to health, oral health, nutrition, child development, mental health, and school readiness, to infants, toddlers, pregnant women, foster care children, children experiencing homelessness, and children eligible for Part C services, under the Individuals for Disabilities Education Act (Zill et al., 2001). EHS home-based programs are recognized as an, “evidenced-based early childhood home visiting service delivery model,” (OPRE, 2020). EHS home-based programs traditionally offer parent-child dyads weekly 90-minute in-person home visits and two in-person socialization sessions a month. The typical structure of in-person visits begins with a greeting followed by individualized child development activities in which the home visitor prompts parent to facilitate the activity. Educator discusses with parents the day’s educational topic, follows up on health needs and requirements, plans for the next visit, and provides community resources and referrals. The educator may perform child development assessments, screenings, or observations. While each visit follows a generalized version of this routine, home visits are individualized to meet families’ needs. The health, nutrition, child development, and other family-focused services are individualized to meet the needs of each child and family (Chazan-Cohen et al., 2012). Parents who have participated in EHS have expressed high satisfaction with services, and children show gains in health and development as a result of their participation (Zill et al., 2001).

EHS services, like many other sectors of health and education who provide home-based services, were drastically changed due to the COVID-19 pandemic. To prevent the spread of the disease, many home-based programs including EHS had to pivot from in-person home visits to tele-home visits. Tele-home visits are generally defined as home visiting services provided through an online video platform that allows families to speak face-to-face with their home visitor. Tele-home visits were a safe way to continue to provide services while following stay-at-home guidelines during the pandemic. Tele-home visits provided a quick solution, yet little was available to identify best practices or effectiveness of services delivered via tele-home visits.

A few telehealth studies focusing on early intervention services for children with a developmental delay indicate virtual home visits can support early learning (Olsen et al., 2012). Utilization of telehealth for these services have shown increased family engagement, improved provider-parent relationship, and increased parenting knowledge, self-efficacy, and empowerment. (Ashburner et al., 2016; Cole et al., 2019; Rooks-Ellis, et al., 2020; Traube et al., 2020). At the same time, other telehealth studies with families and children with developmental delays have identified concerns and limitations including an inability to meet children’s socialization needs, keeping children engaged during the visit, parental hesitancy to lead activities, and limited access to internet and materials (Poole et al., 2022; Yang et al., 2021).

Some evidence, therefore, suggests telehealth visits for early intervention services for children with a known developmental delay, are feasible and provide some strengths and limitations with service delivery. To our knowledge, however, no telehealth studies with a focus on promoting healthy child development among children *without* developmental delays are available to assess whether telehealth visits are also feasible and effective for this large segment of the population. The objectives of our study were to understand parental experiences of replacing EHS in-person home-based services with tele-home visits. More specifically what challenges caregivers faced in using this visit format, parent experiences with services delivered virtually, and overall satisfaction with tele-home visits.

## Methods

### Recruitment Procedure

Parents of children participating in tele-home visits through the EHS home-based programs were recruited via flyers. The Principal Investigator emailed all Head Start Directors in California, requesting their participation in the study. Directors were asked to distribute a recruitment flyer to home visitors in their respective agencies. Home visitors provided the flyer to EHS families. This strategy was used since it is typical for home visitors to provide families with information on community events, announcements, and opportunities. Interested participants were asked to complete an online form or call for more information about the study. Parents were then contacted by two research assistants and were prescreened for eligibility. Three parent inclusion criteria had to be met, (1) enrolled in an EHS home-based program, (2) had previously received in-person home visits, and (3) had transitioned to tele-home visits using any video-chat platform due to the COVID-19 pandemic restrictions. Seventy-eight participants completed the interest form. Forty-four participants were eligible and 35 participated in the focus groups. Of the 25 ineligibles, 15 were receiving phone call visits, take-home packets, or a combination of both during recruitment.

### Focus Groups

Four focus groups were conducted virtually using Zoom. Thirteen mothers participated in English focus groups and 22 mothers participated in Spanish focus groups. Participants received a book and a $10 gift card for their participation. This study was approved by the University Institutional Review Board. The focus groups were conducted based on methods described by Krueger & Casey (2000) with slight modifications to fit a virtual focus group setting. Before starting the questions, confidentiality expectations were reviewed, and participants had the opportunity to leave the group if they no longer wanted to participate. Using a semi-structured guide (*Appendix Table *4), moderators guided the discussion of the focus groups. Parents were asked to describe a typical home visit, including the type of video-chat platform and technology used during visits, child’s engagement, education, relationship with their home visitor, and overall satisfaction. Each focus group had two moderators. The recorded focus groups took place using Zoom and were electronically transcribed verbatim in their original language. Most members of the study team were bicultural and bilingual in English and Spanish. A codebook was developed and refined using consensus-seeking iterative discussion. Coding and analysis were facilitated using Dedoose qualitative software (SocioCultural Research Consultants, Los Angeles, CA, USA). Using an iterative, consensus-seeking inductive content analysis approach (Hsieh & Shannon, 2005), themes were identified related to parents’ experience with the tele-home visits.

## Results

### Participants

The study included 35 participants (referred to as participants, parents, and mothers throughout this text) in focus groups conducted in the summer of 2020. On average, participants reported receiving EHS services for 2 years and receiving services from their current home visitor for 1.7 years. All participants had received at least one month of tele-home visits up to three months, a maximum of 12 tele-home visits. All participants were female with at least one child enrolled in an EHS home-based program. The race and ethnicity of the participants were as follows: 88% were Latino, 6% White, 3% American Indian, and 3% Asian (Table 1). The majority of participants had a high school education or less (76.5%). Approximately 77% of the participants reported an annual household income below $29,999 and 83% were below the federal poverty guidelines. The mean age of the child enrolled in EHS was 28 months and 38% of children received services outside of EHS for special needs. Participants represented a total of eight EHS home-based programs from Southern, Central, and Northern California.

**Table 1:** *top*. Sociodemographic and Early Head Start characteristics

Parent (N = 35)	N (%) or N, std
Age, mean (std)	34, 9.2 std
Gender: Female	(100%)
Race/Ethnicity	
Hispanic/Latino	88%
White	6%
American Indian/Native Alaskan	3%
Asian/Asian American	3%
Highest Level of Education Completed	
Less than high school	29%
High School graduate or GED	47%
2- Year college or some college	15%
4- Year college degree or greater	9%
Marital Status	
Married	57%
Living with partner	11%
Single	31%
Total Household Income	
Less than $19,999	31%
$20,000 to $29,999	46%
Greater than $30,000	23%
Percentage of poverty based on 2020 federal poverty guidelines	
Below Poverty, < 100%	83%
Between 100-130% of Poverty	9%
Over Income, > 130%	9%
**Child (N = 35)**	
Age (months), mean (std)	28, 9.2 std
Gender: Female	50%
Race/Ethnicity	
Hispanic/Latino	89%
White	3%
American Indian/Native Alaskan	3%
Multiracial, 2 or more races/ethnicities	5%
Special Needs, n (%)	13 (38%)
Speech therapy, n	12
Developmental therapy, n	2
Occupational therapy, n	1
ABA therapy, n	1
**Early Head Start Services**	
Length received Early Head Start Services (years), mean (std)	2.0 (1.5)
Length with current home visitor (years), mean (std)	1.7 (1.1)

### Parent Perceptions of Tele-Home Visits

Participants’ experiences with tele-home visits were summarized into five themes with accompanying subthemes. Themes and subthemes are described in further detail below and representative quotes for each are included in Table 2.

**Table 2:** *top.* Parents’ perspectives about tele-home visits—themes and representative quotes

Themes/Subtheme	Selected Illustrative Quotes
**Theme 1: Variability in Initial Parental Attitudes Towards Tele-Home Visits**	
“I [tried] to go into this with an open mind and knowing the dynamic of our visits were going to change overall.“ “I thought at first that this was not going to work because we had to stay in one place, I thought my child was not going to pay attention…but yes this has worked out.”	
**Theme 2: Technology Familiarity, Preferences, and Challenges**	
Variability in Familiarity with Technology	“I found it very difficult. I’m not very tech savvy. I never have been.” *“Pues mi experiencia, también estaba familiarizada en WhatsApp, Messenger y todo. Pero en Zoom la verdad la primera vez. Me costó con la maestra porque a mí me costó un poquito entenderle a la aplicación.”* (In my experience, I was also familiarized with WhatsApp, Messenger and everything. But honestly with Zoom it was my first time. I had difficulty with the teacher because I had difficulty understanding the application.”) “I do Zoom workout classes so she was familiar with zoom…So for her it was really easy to transition.”
Preference for Devices and User Challenges	*“…yo utilizo el celular, pero cuando pasa yo preferiría una pantalla más grande porque cuando la maestra comparte los vídeos o comparte un libro que ella le lee es un difícil para que el niño se enfoque porque es muy pequeña la pantalla como para estar ahí…”* (“…I use a phone, but over time I would prefer a larger screen because when the teacher shares the videos or shares a book that she reads it is difficult for the child to focus since the screen is too small to be there.” “I hear her talking and the words aren’t matching and that just makes it hard to follow.”
**Theme 3: Challenges and Solutions to Child Engagement**	
“Engagement is been- it’s never been consistent. Like one day it went really well and then one day he’s like no, he doesn’t want to or one day he is just kind of in and out. Participate or not so you know I just we just didn’t give up. We just always try. I just went with his flow and like what he wanted.” *“… yo les recomendaría que elijan un espacio en el que su [casa] en el que ellas van, el que se va a tener su visita todas las semanas.”* (“… I would recommend to choose a space in their [home] where they go, where they will have their visit every week.”)	
**Theme 4: Strengths and Limitations of Parental Support and Child Development**	
Increased Parental Self-Efficacy	“I saw that one hour and a half she would come to the home as you know, free babysitting, get away and let her deal with the child, but now I’m actually fully engaged and there’s no choice so -it’s taught me to teach my child, and I think that is priceless.” *“…también prefiero de esta manera…. que sean las visitas por medio de video llamada, y las ventajas que veo en esto que si nos ayuda a involucrarnos más con nuestros hijos en las actividades”* (“I prefer for it to be this way… to have visits by video chat and the advantages I see are that it helps us be more involved with our children in activities.”)
Children Experienced Ongoing Learning and Growth in Child Development	*“Como que, si estuviéramos en una visita regular en casa, leemos, cantamos, realizamos la actividad…Pero realmente no ha cambiado nada de la visita porque logramos hacer completamente todo lo que hacíamos antes.”* (Almost as if we were in a regular home visit, we read, sing, do the activity…But nothing has really changed in the visit because we complete everything we did before.”) “I know that the activities are very relevant to their development and really beneficial for them in any shape or form that they are provided to them.”
Parental Socialization Concerns	“…he’s an only child, so that’s his only way of interacting and having more with other kids, learning how to share, and all that he doesn’t do that at home that much because he’s an only child.” *“…ahorita nadie puede socializar, pero a mi hijo desde muy pequeño ha sido difícil que conviva con otras personas. Siempre lloraba mucho o si se despega un poquito de mí. Pues siento que es la parte difícil en mi caso que pues ya esa actividad pues ya no se va a poder seguir desarrollando.”* (“…right now nobody can socialize, but it has been difficult for my son to be around other people since he was very young. He always cried a lot if he detached a little from me. Well, I feel that this part is difficult in my case that, well, that activity is no longer going to be able to continue developing.”)
**Theme 5: Parent-Home Visitor Relationship**	
Positive Relationship with Home Visitor	*“…también siento que a veces que ya hicimos como que una amistad y a veces le digo mis problemas que están pasando y ella me escucha. A veces me ve llorar y ella me dice, me da la manera de calmarme y me ayuda mucho emocionalmente y muy agradecida con la educadora que me toco.”* (…I also feel that we have created a friendship y sometimes I tell her the problems that are going on and she listens. Sometimes she sees me cry and she tells me, in the calmest manner and she helps me a lot emotionally and I am very grateful with the educator I got.” “…*no cambiaría nada de mi educadora y como dicen las otras compañeras se crea como un vínculo. Más que una maestra, también a la vez es psicóloga, amiga…*” (“…I would not change anything about my educator and as the other mothers said we have created a bond. She is more than a teacher, she is at the same time a psychologist, friend…”
Home Visitor Adaptability Facilitated Transition to Tele-Home Visits	“I like her because she’s really flexible with my son. He doesn’t necessarily always want to do the activity, so she’ll try to get him reengaged with something else… She’s flexible with her time and just trying to give him the education he needs and deserves with the little bit attention span he has.” “If I need to change my time, you know, she’s very accommodating… she’ll change activities based on how my daughter is feeling that day, which I think is really beneficial.”

### Theme 1: Variability in Initial Parental Attitudes Towards Tele-Home Visits

Parents expressed variability in initial attitudes towards tele-home visits. Parents reported feeling uncertain services would be effective, while other parents described being open to trying this new type of service. The majority of parents reported receiving reassurance from their home visitors about the new format of receiving services. The majority of parents who expressed skepticism experienced a change in the perception after receiving services via tele-home visits and reported accepting tele-home visits as a viable method delivery. Parents perceived tele-home visits as a good option to keep children safe during the COVID-19 pandemic.

### Theme 2: Technology Familiarity, Preferences, and Challenges

#### Subtheme: Variability in Familiarity with Technology

Mothers expressed a varying degree of familiarity with the use of virtual platforms, including videoconferencing and social media applications. Three groups were identified, *no experience, some experience*, and *proficient experience* (see Table 2 for parental examples). Mothers with *no experience* reported having difficulty accessing the technology for the first time. Mothers with *some experience* expressed familiarity with at least one social video platform such as FaceTime, Messenger, WhatsApp, or Skype. Mothers who self-identified as *proficient* reported that before COVID-19 they were familiar with various videoconferencing platforms and had ease using it to complete tele-home visits. Parents noted prior use of other virtual platforms made it easier for them to navigate their virtual EHS visits.

### Subtheme: Preference for Devices and User Challenges

Participants reported utilizing three types of devices for tele-home visits: cell phone, tablet, and computer. Mothers most frequently used smartphones due to their mobility, however, mothers stated the smartphone screen was too small and sometimes it was difficult to see what the home visitor was doing. Mothers identified tablets as the best device for tele-home visits due to having a bigger screen as compared to a smart phone and still allowing for the ability to move around the house. When it came to challenges with the technology and devices, mothers reported internet lags, visual and audio problems, limited storage (gigabytes), and difficulty capturing their child’s movements during the virtual visit. Mothers expressed the challenges they perceived during their first tele-home visits resolved over time due to gaining familiarity with the virtual platform, older children assisting, or home visitor’s support. Parents who received step-by-step instruction from their home visitor reported having an easier time utilizing the virtual platform selected by their respective EHS program.

### Theme 3: Challenges and Solutions to Child Engagement

Mothers reported challenges with keeping children engaged during the visit. Common child behaviors during visits included children being easily distracted, running away, refusing to look at the home visitor on the screen, or refusing to sit down. Mothers attributed their child’s disengagement to the child’s short attention span, age, or the child associating screen time with entertainment. Children’s prior familiarity with smart phones was either a barrier or promoter of engagement. If children had exposure to the parent using video for communicating with family or friends it was a promoter. If the use of mobile devices were used for leisure and playing games then children wanted to play with the device during the visits.

Many mothers, however, also reported adopting strategies over time to keep their child engaged in tele-home visits. Development of a routine that included constant reminders, having a designated space in the home for the visits, or playing a specific-song before each visit supported the child’s transition to tele-home visit activities. Designated learning spaces included, a carpet or learning corner free of distractions. Parents also reported allowing their children to leave the learning space and return when ready to continue. Some parents commented the screen was a distractor for their child and having the screen off was beneficial in keeping their child engaged. When this strategy was used, the home visitor provided the guidance and coaching via audio while the child development activity was completed at home.

Parents also reported the importance of having flexibility with the typical order of activities and duration of a home-visit in order to follow the child’s lead in engagement and participation. The parent and home visitor adapted the typical structure of a home visit by continuously interchanging between child development activities and parent discussions. When the child showed interest, parents took the opportunity to engage the child in activities. If children provided cues they needed a rest, then the mother and home visitor would transition to discussing a topic relevant to the home visit. When asked about experiences with the duration of time to complete the weekly telehealth visit, most mothers indicated one hour would be an ideal time for the tele-home visit length, with 30–45 min dedicated to child development activities. Many stated the length of time depended on how their child was adapting to tele-home visits and appreciated the flexibility and adaptability to receive both parent and child content during visit.

### Theme 4: Strengths and Limitations of Parental Support and Child Development

#### Subtheme: Increased Parental Self-Efficacy

Mothers shared tele-home visits prompted them to take an increasingly active and hands-on role as their child’s “first teacher” and observer in their child’s learning and tele-home activities. Parents reported higher degrees of engagement and participation in child development activities than in-person home visits. Parents also reported an increased level of engagement incorporating child development activities into their daily routines. Some mothers also expressed that children were learning more as a result of their increased interaction. The success of increased parent participation was facilitated by joint planning with the home visitor and prepping materials for the activity of the tele-home visit.

### Subtheme: Children Experienced Ongoing Learning and Growth in Child Development

Participants perceived the child-development focused activities delivered via tele-home visits as structured, individualized, and effective in supporting school readiness for their child. Children were perceived to continue learning and advancing in their milestones. Most parents mentioned the activities they did during tele-visits were consistent with in-person activities. Typically, they would start with singing a song, reading a book, and then completing activities. Mothers also described the hands-on activities children enjoyed the most such as crafts, cooking, and outdoor and physical activities. The variability in the educational resources provided to EHS participants and was contingent upon each program. Some mothers stated they received activities, supplies, books, e-books, curriculum manuals, and other resources. Those who received these resources found it helpful and supported being able to implement the child development activities at home.

### Subtheme: Parental Socialization Concerns

Mothers voiced concerns about tele-home visits not meeting their child’s socialization needs. Some mothers reported their children missed interacting with their home visitors in person. Mothers suggested having children engage with other children via Zoom to continue their social development. Although mothers expressed a preference for in-person home visits to promote child social skills they also expressed uncertainty of having in-person visits due to concerns about exposing other families and being exposed to COVID-19.

### Theme 5: Parent-Home Visitor Relationship

#### Subtheme: Positive Relationship with Home Visitor

All parents described a supportive relationship with their home visitors. Parents were able to cope with the transition to tele-home visits with the help of their home visitor. Mothers were challenged to access and use technology unfamiliar to them, troubleshoot and cope with the nuances of virtual home visits. In addition, mothers who were experiencing a sense of grief and loss due to illness, deaths, and economic hardships of the pandemic, reported that their primary support agent was their assigned home visitor. Many mothers associated the positive relationship with the multiple roles home visitors had during and outside tele-visits, including providing families with educational and family resources to support their family’s experience during these challenging times.

### Subtheme: Home Visitor Adaptability Facilitated Transition to Tele-Home Visits

Mothers stated their home visitor was flexible to both child and parent needs. Parents expressed that their home visitor made several attempts to engage children and parents through a set of trial-and-error strategies. Mothers expressed home visitor’s creativity with activities and their persistence were vital to their adaptation to tele-home visits and continued learning for children. In addition, mothers stated home visitors’ flexibility with meeting days and times was critical as many mothers experienced an increase load of in- and out-of-home work.

## Discussion

In this study, we explored parental perceptions of EHS tele-home visits during the COVID-19 pandemic. Parent perceived successes included increased parental engagement, uncompromised learning and developmental growth for their child, and a positive parent-home visitor relationship. Parents reported taking a more active role with EHS visits and described joint planning with home visitors, becoming the primary teacher for the child-development focused activities, and becoming keen observers of developmental milestones. Parents reported benefiting from a variety of child activities, supplies, and resources provided by home visitors, including the real-time emotional support and linkages to community resources home visitors provided as families experienced adversity during the COVID-19 pandemic.

Our results align with previous studies which indicate a tele-home service delivery model can increase parental engagement and is also dependent on the degree of parental engagement and participation (Brookes et al., 2006; Rooks-Ellis et al., 2020). While we found varying degrees of child engagement with telehealth visits, all parents reported their child was able to continue learning. The learning was attributed in large part to parent’s perceived increased engagement in the child-development activities, flexibility with the “order” of the content and activities used in a typical in-home visit (being able to continuously move from parent- to child-focused content dictated by child’s attention span and interest), and being able to adapt the visit length. Our findings suggest the parent-home visitor relationship is critical to build parent engagement during virtual visits. Parents described being proactive facilitators of planned and spontaneous activities for their child, thereby fostering individualized learning experiences. Although there was an overwhelming preference for in-person home visits over tele home visits, most parents were not willing to welcome their home visitor into their homes until the COVID-19 virus was contained.

Challenges with tele home visits, included the availability of an internet connection, a device, and the proficiency necessary to use the technology available. We found many families had access to the internet through their smartphones, but many lacked the knowledge and skill necessary to download, install, or use applications, or navigate through a series of websites to gain information. The “digital divide” where some individuals are advantaged by the internet while others are relatively disadvantaged by the internet (Rogers, 2001), was palpable as families transitioned to telehealth visits. The “digital divide” challenges, however, were overcome with time and assistance from the home visitors. Parents noted individualized training and troubleshooting with their home visitors were particularly effective. EHS programs, therefore, are in an ideal position to narrow the digital gap through parent training and coaching families to access the technology needed. Families served by EHS would benefit from knowing how to use these new virtual communication portals. In this study, the transition to tele-home visits encouraged many parents to learn to use technology as they had not before and prepared parents to possess the necessary skills to use beyond EHS.

Another finding was the lack of peer-to-peer socialization activities for children as a result of the tele-home visits. Many parents reported that their child had not interacted with other children for months due to COVID-19 restrictions. Similar findings were reported by Soltero-González & Gillanders (2021), where parents were concerned with the prolonged social distancing impacting on their children’s socio-emotional health, and researchers stressed the importance of educators being responsive to children’s interests. The socialization needs are particularly relevant to several vulnerability factors including developmental age, previous mental health conditions, educational and socioeconomic status, and being quarantined (Singh et al., 2020). Despite these socialization limitations, parents expressed positive tele-home visit experiences and an ability to continue child-focused activities that supported positive parenting and healthy parent-child attachment.

### Recommendations

Table 3 summarizes recommendations based on parents’ suggestions that other family agencies may find helpful as they transition to telehealth visits, as many parents and families may have variability in their knowledge and proficiency in using smartphones or video-chat platforms. To address challenges in technology, programs are recommended to consider a technology needs assessment with questions about access and familiarity, and consider providing parents with devices and remote internet connections for families who do not have access to technology. As we move forward past the pandemic, likely, many of the systems and practices developed as a response to the pandemic will stay and require further use of the internet and technology to deliver services previously accessed in person. We recommended that programs evaluate their program’s technology proficiency including a technology assessment of parents and all staff working with families in a virtual format.

**Table 3:** *top.* Early Head Start tele-home visit recommendations

Recommendations
**Pre-Tele Home Visit Expectations**
• Programs are recommended to first consider engaging in a discussion with families to explain the new delivery service model, including setting expectations, providing program and child goals, and explaining parent and home visitor roles in tele-home visits.
**Technology**
• **Home Visitor Technology Training -** Programs may need to evaluate their home visitors’ technology strengths and areas of need, and in response provide trainings. • **Families’ Technology Accessibility** o Ensure families have access to quality connectivity – Families with connectivity issues may benefit from hotspot devices such as a Mi-Fi device or referral to low-cost internet programs such as Lifeline. o Verify the availability of a device – Some families may only have one device that is shared by several children. Families may benefit from being provided an additional device. Parents’ recommended device was a tablet due to its portability and larger screen. • **Parents’ Technology Familiarity –** Home visitors may need to tailor individualized training, troubleshoot with families prior to first tele-home visit, and provided step-by-step guides.
**Child Engagement and Learning**
• **Designated Learning Space** – We recommended home visitors work with families to identify and set up a consistent learning space. Ask families to select a place in their home to do tele-home visits and develop a routine to indicate the start of the visit. • **Joint Planning –** Home visitors and parents should cooperatively plan visits to individualize curriculum and help home visitors provide resource recommendations. • **Child Development Activities** – Home visitors should experiment with diverse child development activities and identify child’s areas of interest. • **Resources –** Provide parent handouts, curriculum manuals, and materials that support the curriculum activities. • **Visit Length –** Start by conducting 30minute tele home visits and follow the lead of each family to gradually build up to 1 hour. Some families may need visits to be split into two-fifteen-minute sessions and slowly build up to twohalf-hour sessions each week.
**Sample Tele-Home Visit Schedule**
1. Begin with a song to mark the beginning of the home visits 2. Greet parent and child (1–5 min) 3. Health check (5 min) – ask about the health of the parent and child 4. Transition to reviewing the goal of the visit and the child development activity (1–5 min) 5. Explain the child development activity (5 min) 6. Practice parent-child activity (10–20 min) 7. Discuss observation of child’s skills with parent (5 min) 8. Transition to parent discussion (15–20 min or end if family is getting uneasy) 9. Plan for next visit and identify any resources needed (5 min) 10. Good-bye ritual 11. Immediately document home visit content

To address the lack of socialization opportunities with virtual home visits, Home visitors can assess child’s socialization needs and identify those at highest-risk in order to guide limited resources to support socio-emotional development during the pandemic. Feasible recommendations made by parents in this study included: (a) Zoom sessions with other EHS children; (b) virtual support groups; (c) virtual home visits with groups of 2–3 children with interactive activities planned. In addition, when it deemed safe, it is recommended programs plan socially distanced activities outdoors. In the path to the post-COVID-19 pandemic, we anticipate that virtual home visits may increase access to families reluctant to resume in-person services, immunocompromised families, families who live in rural areas, inconvenient distances from home visitors, migrant families, and families experiencing homelessness. Programs may consider tele-home visits as an option during any public health emergency. Virtual delivery can be adapted to meet families’ individualized needs including families in search of hybrid visits because of work schedules and not being able to participate in weekly in-person visits. Programs may have fewer cancellations due to the flexibility of scheduling a virtual visit. Families who had a COVID-19 exposure or other contagious disease may receive a virtual visit without having to lose their weekly home visit. Families who do not have a means of transportation for group socialization or education sessions usually held at EHS sites may be able to participate virtually. The success perceived by the parents in the study was attributed to their relationship with their home visitor, access to technology, educational material being provided including curriculum manuals, supplies, and technology devices, flexibility with the length of the home visits, and parental engagement.

## Limitations

Our results may not be generalizable to other EHS centers or similar type of programs as our results were based on a group of parents from a limited number of EHS programs. The sample included English and Spanish-speaking participants from Southern, Central, and Northern California. The timing of our results may have also affected our findings as the data was collected at the beginning of the pandemic when parents had received approximately 3–4 months of tele-home visits. EHS programs differed regarding how quick programs transitioned to tele-home visits. We estimate that between the start of stay-at-home orders and when focus groups were conducted families completed a maximum of 12 tele-home visits. Due to the immediate transition to tele-home visits, the opportunity to complete a technology needs assessment may not have been available for everyone. Potentially, after 12-months into the pandemic, many families have gained digital knowledge and proficiency. This study reflects the perspectives of parents who had experienced in-person sessions with a home visitor which may have provided the opportunity to build a relationship and trust before transitioning to virtual visits; therefore, perceptions may not be generalizable to parents who never connected with a home visitor in-person. Additional studies are needed to determine whether similar parental perceptions could be established without any prior in-person visits. We did not collect parental perceptions on other virtual visit types, such as phone or audio only visits. Finally, this study was not designed to measure child outcomes, such as developmental milestone advancement, but is of interest for a future study in order to evaluate for gains or losses of milestones pre- and post-pandemic (i.e. in-person versus telehealth).

## Conclusion

Our findings suggest tele-home visits have the potential to be a viable service delivery method for EHS home-based programs. Optimizing tele-home experiences for both staff and families may include providing parental materials for creating an engaging environment in the child’s home, supplies and resources for child-focused activities that promote learning and development, and by providing parents with needed technological training and support. The tele-home visit delivery model will likely continue to be critical during the COVID-19 pandemic, and post pandemic. Thus, it is critical to develop effective parent engagement during tele-home visits, in partnership withthe family’s assigned home visitor and EHS program.

## Data Availability

Data will be available upon request.

## Appendix

**Table 4:** Tele-home visits focus group question guide

Perceptions before the first visit.
1. What did you think about tele-home visits before you tried them? PROBE: How did you imagine tele-home visits would be?
**Technology**
1. Before your first tele-home visit were you familiar with video chat in any platform?
2. Tell me what you had to do to get set up for the tele-home visit. PROBES: How hard or easy was it for you to get set up/prepare? (download zoom, become familiar)?
3. Do you use social media? If so tell me about how you use it. PROBE: How often do you use Facebook, Twitter, YouTube, and Zoom
4. What device have you used for the tele-visits (in phone, tablet, computer, smart tv, other device)? PROBE: What devices do you have at home? What device is ideal?
5. Have you had any challenges with tele-home visits? PROBE: Have you experienced any technology barriers, such as video quality, poor sound, internet is slow.
6. What resources do you think a family may need to participate in tele-home visits?
**Engagement**
1. How did your child react to the transition to tele-home visits? PROBE: Did your child engage in the tele-home visit activities?
2. For how long is your child engaged in the tele-home visit?
3. What did you think about the visit length? PROBE: What would be the ideal length of the tele-home visit?
4. Can you describe what you do in a tele-home visit from start to finish? PROBE: Child development activities, receive emotional support, get resources, health information, etc.
5. Do you receive any resources? PROBE: Do you find the resources you receive are useful?
**Education**
1. How would you describe the part of the tele-home visit designed to help with your child’s development? PROBE: Do you feel that the child development activities covered in the tele-home visits are helping your child be school ready? Do you feel your child is learning with the tele-home visits?
2. What activities would you like to see?
3. What do you like about the tele-home visits? What do you dislike about the tele-home visits?
4. What components of the tele-home visits do you feel are effective? Probe (1) Educational services-learning activities to do at home to get child school ready; (2) Mental Health support, receive emotional support through home visitor or counselor or learning how to support children’s emotional well-being; (3) Family services-learning about resources available in community; (4) Disability Services-receiving support in making sure child is getting services through Regional Center; (5) Health-learning about health and how to keep child healthy; (6) Nutrition-learning about healthy nutrition, (7) Safe environments-learning how to keep child safe; (8) anything other component that is effective that was not mentioned.
**Parent-Home Visitor Relationship**
1. How long has your home visitor been assigned to you?
2. How would you describe your relationship with your home visitor? PROBE: What are the qualities you like best about your home visitor? What are the qualities you like the least about your home visitor?
3. What activities do you enjoy engaging in with your home visitor?
4. How do you feel about not having your home visitors come to your home?
5. Was your child’s father involved in the regular in person home visits? Is the father engaged in tele-home visits?
**Overall**
1. Overall, how do you feel about the tele-home visits?
2. Would you be willing to use tele-home visits again?
3. Is there anything else you would like us to know about tele-home visits?

## References

[CR1] Ashburner J, Vickerstaff S, Beetge J, Copley J (2016). Remote versus face-to-face delivery of early intervention programs for children with autism spectrum disorders: Perceptions of rural families and service providers. Research in Autism Spectrum Disorders.

[CR2] Brookes SJ, Summers JA, Thornburg KR, Ispa JM, Lane VJ (2006). Building successful home visitor–mother relationships and reaching program goals in two Early Head Start programs: A qualitative look at contributing factors. Early Childhood Research Quarterly.

[CR3] Chazan-Cohen R, Halle TG, Barton LR, Winsler A (2012). Supporting optimal child development through Early Head Start and Head Start programs: Reflections on secondary data analyses of FACES and EHSREP. Early Childhood Research Quarterly.

[CR4] Cole B, Pickard K, Stredler-Brown A (2019). Report on the Use of Telehealth in Early Intervention in Colorado: Strengths and Challenges with Telehealth as a Service Delivery Method. International Journal of Telerehabilitation.

[CR5] Fenichel E, Mann TL (2001). Early Head Start for low-income families with infants and toddlers. The Future of Children.

[CR6] Hsieh HF, Shannon SE (2005). Three approaches to qualitative content analysis. Qualitative Health Research.

[CR7] Krueger RA, Casey MA (2000). Focus groups: A practical guide for applied research.

[CR8] Olsen S, Fiechtl B, Rule S (2012). An Evaluation of Virtual Home Visits in Early Intervention: Feasibility of “Virtual Intervention". Volta Review.

[CR9] OPRE. (2020). *Early childhood home visiting models: Reviewing evidence of effectiveness*. (Report No. 2020 – 126). Administration for Children and Families, Office of Planning, Research, and Evaluation, U.S. Department of Health and Human Services. https://www.acf.hhs.gov/opre/report/home-visiting-evidence-effectiveness-review-brief-december-2020

[CR10] Poole ME, Fettig A, McKee RA, Gauvreau AN (2022). Inside the Virtual Visit: Using Tele-Intervention to Support Families in Early Intervention. Young Exceptional Children.

[CR11] Rogers EM (2001). The Digital Divide. Convergence.

[CR12] Rooks-Ellis, D., K. Howorth, S., Kunze, M., Boulette, S., & Sulinski, E. (2020). Effects of a parent training using telehealth: Equity and access to early intervention for rural families. *Journal of Childhood, Education & Society, 1*(2), 141–166. https://doi.org/10.37291/2717638X.20201242

[CR13] Singh S, Roy D, Sinha K, Parveen S, Sharma G, Joshi G (2020). Impact of COVID-19 and lockdown on mental health of children and adolescents: A narrative review with recommendations. Psychiatry research.

[CR14] Soltero-González, L., & Gillanders, C. (2021). Rethinking home-school partnerships: Lessons learned from Latinx parents of young children during the COVID-19 era. *Early Childhood Education Journal*, *49*, 965–976. 10.1007/s10643-021-01210-4 10.1007/s10643-021-01210-4 PMC8169385 34092996

[CR15] Traube DE, Hsiao HY, Rau A, Hunt-O’Brien D, Lu L, Islam N (2020). Advancing Home Based Parenting Programs through the Use of Telehealth Technology. Journal of Child and Family Studies.

[CR16] Yang HW, Burke M, Isaacs S, Rios K, Schraml-Block K, Aleman-Tovar J, Tomkins J, Swartz R (2021). Family Perspectives toward Using Telehealth in Early Intervention. Journal of Developmental and Physical Disabilities.

[CR17] Zill, N., Resnick, G., Kim, K., McKey, R. H., Clark, C., Pai-Samant, S., Connell, D., Vaden-Kiernan, M., Obrien, R., & D’Elio, M. A. (2001). *Head Start FACES: Longitudinal findings on program performance: Third progress report*. Administration of Children, Youth, and Families. https://files.eric.ed.gov/fulltext/ED453969.pdf

